# Supplementary material for: Upstream watershed condition predicts rural children’s health across 35 developing countries
Source: Nat Commun. 2017 Oct 9;8:811. doi: 10.1038/s41467-017-00775-2 (PMC5634511; doi:10.1038/s41467-017-00775-2)
Supplement: Supplementary file 1 — Supplementary Information [file 41467_2017_775_MOESM1_ESM.pdf]

Supplementary Table 1: Coefficients from mixed effects logit models

	(1) All clusters	(2) Rural	(3) Urban	(4) Rural Unimproved water	(5) Rural Improved water
Fixed effects variables					
Age (months)	-0.0303	-0.0299	-0.0313	-0.0286	-0.0355
	(0.0004)	(0.0005)	(0.0008)	(0.0006)	(0.0012)
Wealth*	-0.172	-0.136	-0.285	-0.139	-0.155
	(0.0187)	(0.0256)	(0.0296)	(0.0295)	(0.0512)
Education*	-0.163	-0.153	-0.204	-0.161	-0.117
	(0.0184)	(0.0247)	(0.0287)	(0.0292)	(0.0476)
Improved sanitation*	-0.0730	-0.0523	-0.177	-0.0222	-0.118
	(0.0183)	(0.0223)	(0.0337)	(0.0251)	(0.0485)
Improved water*	-0.00302	-0.0810	0.0476	-	-
	(0.0197)	(0.0271)	(0.0318)	-	-
Precipitation t-1	-0.203	-0.218	-0.178	-0.228	-0.190
	(0.0120)	(0.0158)	(0.0188)	(0.0173)	(0.0364)
Precip t – Precip t-1	-0.0514	-0.0572	-0.0341	-0.0624	-0.0480
	(0.0213)	(0.0277)	(0.0338)	(0.0302)	(0.0629)
Temperature t-1	0.0757	0.0588	0.0805	0.0194	0.153
	(0.0109)	(0.0138)	(0.0183)	(0.0160)	(0.0259)
Temp t – Temp t-1	-0.0143	-0.0806	0.123	0.0621	-0.297
	(0.0310)	(0.0389)	(0.0518)	(0.0468)	(0.0708)
Upstream human activity	0.0836	0.0916	0.0622	0.114	0.0723
	(0.0242)	(0.0314)	(0.0382)	(0.0349)	(0.0666)
Upstream tree cover	-0.133	-0.162	-0.0395	-0.169	0.104
	(0.0449)	(0.0571)	(0.0735)	(0.0676)	(0.104)
Constant	-1.562	-1.587	-1.391	-1.601	-1.604
	(0.0212)	(0.0246)	(0.0470)	(0.0266)	(0.0606)
Random effects variance components					
Cluster level variance	0.688	0.701	0.635	0.704	0.740
	(0.0202)	(0.0244)	(0.0355)	(0.0272)	(0.0644)
Household level variance	1.603	1.530	1.800	1.399	2.087
	(0.0534)	(0.0614)	(0.108)	(0.0652)	(0.172)
Number of children	293,362	197,454	95,908	155,232	42,222
Number of clusters	23,087	12,518	10,569	10,624	5,857

*Binary variable.
Standard errors in parentheses.

Supplementary Table 2: Difference in mean characteristics between urban and rural households

Variable	Urban	Rural	Diff (Urban - Rural)
High education (%)	56%	21%	35%***
High wealth (%)	61%	18%	43%***
Improved sanitation (%)	74%	36%	38%***
Improved water (%)	63%	22%	41%***
Precipitation (mm)	126	125	0.6
Temperature (degrees Celsius)	22.8	22.7	0.1
Upstream human activity (%)	37%	31%	6%***
Upstream tree cover (%)	8%	10%	-2%***

*** P>|z| = 0.000

Supplementary Table 3: Coefficients from mixed effects logit models.

	(1)<br>Rural – High human activity upstream	(2)<br>Rural – Low human activity upstream
Fixed effects variables		
Age (months)	-0.0264	-0.0314
	(0.0009)	(0.0006)
Wealth*	-0.0688	-0.158
	(0.0486)	(0.0301)
Education*	-0.0857	-0.170
	(0.0463)	(0.0294)
Improved sanitation*	0.0306	-0.0930
	(0.0406)	(0.0269)
Improved water*	-0.0740	-0.0783
	(0.0530)	(0.0316)
Precipitation t-1	-0.211	-0.208
	(0.0306)	(0.0187)
Precip t – Precip t-1	-0.0378	-0.0713
	(0.0476)	(0.0357)
Temperature t-1	0.0604	0.0489
	(0.0283)	(0.0159)
Temp t – Temp t-1	-0.193	-0.0568
	(0.105)	(0.0426)
Tree cover 25th-50th percentile	0.126	-0.0239
	(0.115)	(0.0625)
Tree cover 50th-75th percentile	-0.0176	0.0803
	(0.115)	(0.0597)
Tree cover > 75th percentile	-0.258	-0.209
	(0.102)	(0.0642)
Constant	-1.672	-1.534
	(0.0512)	(0.0275)
Random effects variance components		
Cluster level variance	0.797	0.662
	(0.0495)	(0.0280)
Household level variance	1.516	1.541
	(0.113)	(0.0733)
Number of children	59,508	137,946
Number of clusters	4,001	8,672

*Binary variable.
Standard errors in parentheses.

Supplementary Figure 1: Factors associated with DD in urban and rural areas of West and Central Africa (10 countries). Odds ratios from mixed effects logit models and 95% CI show that in this subset of countries the effects of tree cover upstream are non-significant in urban areas, while these are significant and negative (reduce the probability of DD) in rural areas.

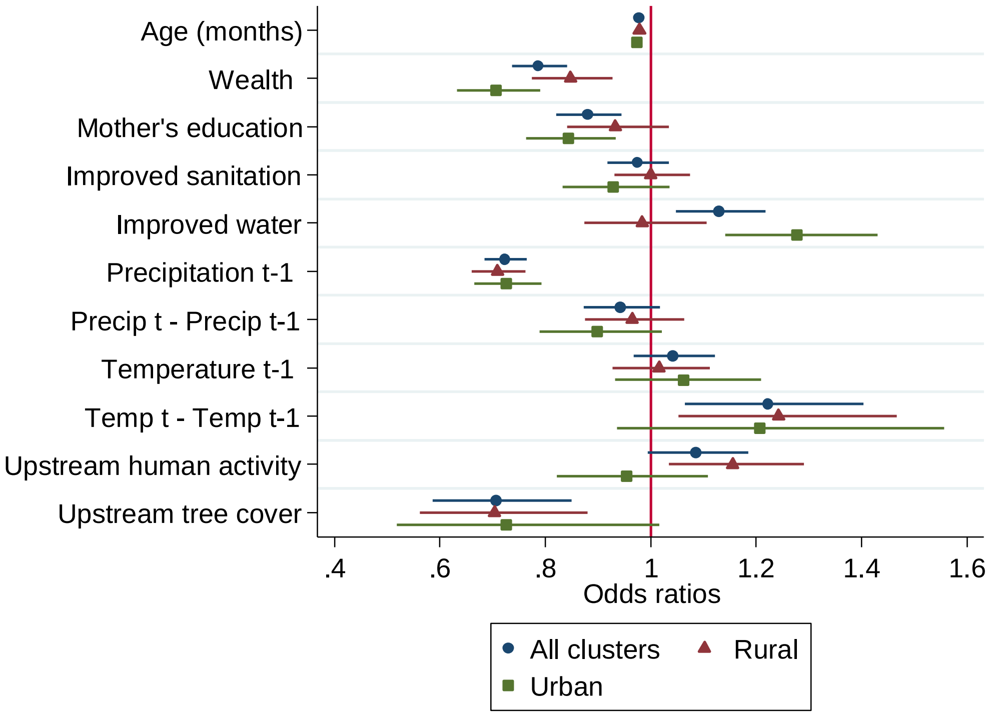

Supplementary Table 4: Mean socioeconomic characteristics for 35 countries in our sample. Individual and household level variables.

Country	DHS year	Number of observations	% Rural	% with diarrhea*	Mean age in years	% Mothers with secondary or higher education	% High wealth	Mean time to water (min)	% improved water	% improved sanitation
Bangladesh	2011	7,515	69%	5%	3	49%	39%	5	11%	54%
Burkina Faso	2010	12,787	77%	15%	2	6%	39%	20	19%	2%
Cameroon	2011	10,308	60%	20%	2	34%	34%	21	33%	52%
Colombia	2010	17,083	36%	14%	3	66%	18%	2	66%	86%
Congo DR	2007	7,674	59%	16%	2	34%	38%	31	26%	60%
Dominican Republic	2007	10,468	44%	16%	3	49%	22%	4	30%	90%
Egypt	2008	9,840	64%	9%	2	61%	35%	1	92%	99%
Ethiopia	2005	8,784	86%	17%	2	7%	38%	45	24%	10%
Gabon	2012	5,564	38%	17%	2	54%	18%	23	65%	35%
Ghana	2008	2,721	66%	20%	2	38%	29%	18	32%	54%
Guinea	2005	5,362	78%	15%	2	5%	33%	17	17%	24%
Haiti	2005	5,318	63%	22%	2	23%	31%	24	33%	53%
Indonesia	2002	14,411	59%	10%	2	50%	32%	12	20%	58%
Jordan	2007	9,753	33%	16%	2	87%	19%	NA	80%	99%
Kenya	2008	5,602	76%	17%	2	22%	37%	30	28%	41%
Lesotho	2009	3,389	83%	12%	2	38%	29%	20	58%	26%
Liberia	2007	5,168	65%	20%	2	15%	30%	11	5%	21%
Madagascar	2008	11,434	82%	8%	2	20%	32%	17	23%	3%
Malawi	2010	17,676	91%	17%	2	14%	32%	30	20%	9%
Mali	2006	12,312	70%	12%	2	6%	39%	8	23%	77%
Moldova	2005	1,472	46%	8%	2	99%	49%	NA	43%	80%
Morocco	2003	5,771	57%	12%	2	17%	28%	NA	57%	75%
Mozambique	2011	10,143	68%	10%	2	15%	43%	36	35%	25%
Namibia	2006	4,536	63%	12%	2	57%	34%	14	72%	36%
Nepal	2011	4,774	80%	13%	3	33%	30%	9	48%	46%
Nigeria	2008	25,260	73%	11%	2	28%	31%	19	9%	48%
Philippines	2008	6,182	58%	9%	2	72%	27%	5	33%	78%
Rwanda	2010	8,402	86%	13%	3	9%	36%	34	31%	73%
Senegal	2010	11,128	70%	19%	2	7%	23%	13	54%	77%
Swaziland	2006	2,397	75%	14%	2	56%	39%	21	50%	79%
Tanzania	2010	6,961	82%	14%	2	11%	35%	31	34%	9%
Timor-Leste	2009	9,223	77%	15%	2	37%	36%	18	46%	48%
Uganda	2011	7,128	78%	23%	2	22%	37%	43	20%	11%
Zambia	2007	5,705	68%	15%	2	24%	33%	18	25%	29%
Zimbabwe	2010	4,871	71%	13%	2	65%	37%	19	29%	56%

Supplementary Table 5: Mean environmental characteristics for 35 countries in our sample. Cluster level variables.

Country	Temperature (deg Celcius) during survey month	Precipitation (mm) during survey month	Upstream Livestock and Population index	Upstream tree cover index
Bangladesh	26	237	58%	76%
Burkina Faso	27	95	40%	14%
Cameroon	25	152	18%	2%
Colombia	23	194	70%	2%
Congo DR	23	63	14%	0%
Dominican Republic	26	126	46%	64%
Egypt	20	2	21%	34%
Ethiopia	19	85	20%	12%
Gabon	26	215	28%	0%
Ghana	26	128	38%	4%
Guinea	28	86	30%	10%
Haiti	24	99	26%	62%
Indonesia	26	260	25%	0%
Jordan	24	0	0%	11%
Kenya	22	73	12%	0%
Lesotho	16	93	0%	0%
Liberia	26	89	2%	0%
Madagascar	24	236	64%	0%
Malawi	19	9	0%	0%
Mali	28	124	51%	35%
Moldova	20	70	4%	0%
Morocco	12	64	47%	11%
Mozambique	22	26	0%	0%
Namibia	24	83	11%	0%
Nepal	15	23	5%	70%
Nigeria	26	206	54%	5%
Philippines	26	297	13%	30%
Rwanda	19	111	32%	0%
Senegal	26	2	0%	49%
Swaziland	20	90	20%	0%
Tanzania	24	142	51%	0%
Timor-Leste	24	126	41%	0%
Uganda	22	107	24%	0%
Zambia	19	11	0%	0%
Zimbabwe	22	110	32%	0%

Supplementary Table 6: Definition of improved/unimproved sanitation and water based on DHS codes and JMP classification. Percentage of observations in each category.

Improved water	% of obs	Improved sanitation	% of obs
DHS Code 11, 12, 13, 14<br>Piped into dwelling or yard, tap, neighbor's tap, public tap	36%	DHS Code 11, 12, 13 , 14 and 21<br>Flush toilet, piped, flush to septic tank or pit latrine, in house or shared, ventilated/improved pit latrine, latrine with slab	48%
**Unimproved water**	**% of obs**	**Unimproved sanitation**	**% of obs**
DHS Code 21, 22, 23, 24, 25, 31, 32, 33, 34, 35, 36 and 41<br>Unprotected well/spring, tubewell or borehole, well in dwelling or public spring	31%	DHS Code 22, 23, and 24<br>Traditional pit latrine (flush somewhere else, no slab, unventilated/unimproved)	22%
DHS Code 41, 42, 43, 44 and 51<br>Surface water, river, dam, lake, pond, stream, canal, rainwater	29%	DHS Code 31 and 32<br>No facility (bush, field, river, canal, ocean)	27%
DHS Code 61, 62, 64, 71, 72, 81<br>Water vendors, water in plastic bags, other	4%	DHS Code 41, 42, 43, 44 and 96<br>Hanging toilet/latrine, bucket, other	2%

Supplementary Figure 2: The relationship between the country average of the predicted probability of diarrhea and the country average of the cluster-level random effect is positive and shows a linear fit which suggest that the cluster-level random effect is effectively controlling for country-level variation in the probability of diarrhea.

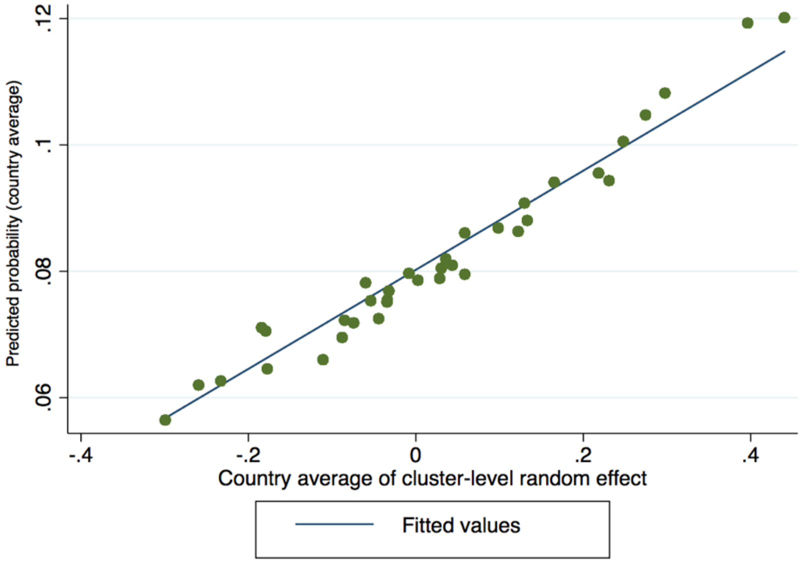

Supplementary Figure 3: Factors associated with DD in urban and rural areas (full model). Odds ratios from mixed effects logit models and 95% CI show that the effects of tree cover upstream are non-significant in urban areas, while these are significant and negative (reduce the probability of DD) in rural areas with tree cover above the 75th percentile.

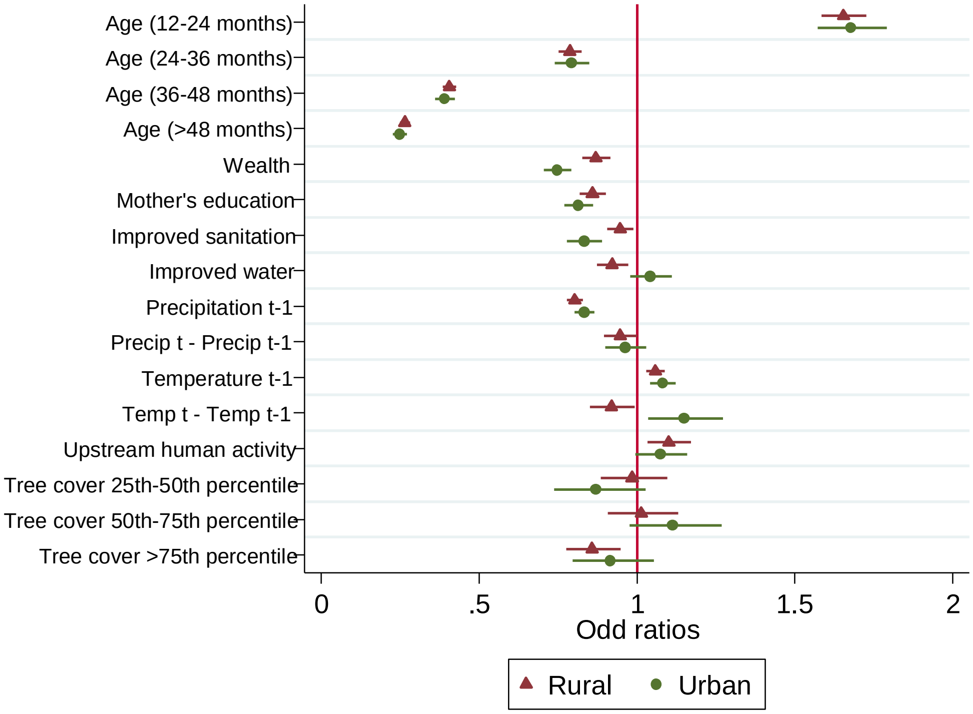

Supplementary Table 7. Pairwise correlation coefficients for the variables in the model. Correlation coefficients are lower than 0.4 which show low to moderate correlation across variables. Correlations at the cluster and household level are addressed by the random effects model. P-values in parentheses.

	Age	Wealth	Education	Improved sanitation	Improved water	Precipitation t-1	Precip t - Precip t-1	Temperature t-1	Temp t - Temp t-1	Upstream human activity	Upstream tree cover
Age	1	-	-	-	-	-	-	-	-	-	-
Wealth	0.00<br>(0.03)	1	-	-	-	-	-	-	-	-	-
Education	-0.02<br>(0.00)	0.29<br>(0.00)	1	-	-	-	-	-	-	-	-
Improved sanitation	0.01<br>(0.00)	0.24<br>(0.00)	0.34<br>(0.00)	1	-	-	-	-	-	-	-
Improved water	0.01<br>(0.00)	0.27<br>(0.00)	0.27<br>(0.00)	0.31<br>(0.00)	1	-	-	-	-	-	-
Precipitation t-1	0.01<br>(0.00)	0.01<br>(0.00)	0.07<br>(0.00)	-0.02<br>(0.00)	-0.17<br>(0.00)	1	-	-	-	-	-
Precip t - Precip t-1	-0.01<br>(0.00)	-0.03<br>(0.00)	0.03<br>(0.00)	0.04<br>(0.00)	0.03<br>(0.00)	-0.26<br>(0.00)	1	-	-	-	-
Temperature t-1	-0.01<br>(0.00)	-0.01<br>(0.00)	-0.03<br>(0.00)	0.01<br>(0.00)	-0.17<br>(0.00)	0.29<br>(0.00)	0.05<br>(0.00)	1	-	-	-
Temp t - Temp t-1	0.00<br>(0.01)	0.00<br>(0.02)	0.09<br>(0.00)	0.05<br>(0.00)	0.09<br>(0.00)	-0.08<br>(0.00)	-0.01<br>(0.00)	-0.16<br>(0.00)	1	-	-
Upstream human activity	0.00<br>(0.11)	-0.03<br>(0.00)	-0.01<br>(0.00)	0.02<br>(0.00)	-0.04<br>(0.00)	0.40<br>(0.00)	0.16<br>(0.00)	0.16<br>(0.00)	-0.08<br>(0.00)	1	-
Upstream tree cover	0.01<br>(0.00)	-0.01<br>(0.00)	-0.05<br>(0.00)	0.11<br>(0.00)	0.01<br>(0.00)	0.01<br>(0.00)	-0.24<br>(0.00)	0.18<br>(0.00)	0.02<br>(0.00)	-0.03<br>(0.00)	1
